# Technical report: a high-dose-rate interstitial brachytherapy boost for residual sinonasal undifferentiated carcinoma

**DOI:** 10.1093/jrr/rrac054

**Published:** 2022-09-22

**Authors:** Yusaku Miyata, Naoya Murakami, Yoshitaka Honma, Taisuke Mori, Seiichi Yoshimoto, Tairo Kashihara, Mihiro Takemori, Yuko Nakayama, Jun Itami, Etsuyo Ogo, Hiroshi Igaki

**Affiliations:** Department of Radiation Oncology, National Cancer Center Hospital, 5-1-1 Tsukiji, Chuo-ku, Tokyo 104-0045, Japan; Department of Radiology, Kurume University School of Medicine, 67 Asahimachi, Kurume city, Fukuoka 830-0011, Japan; Department of Radiation Oncology, National Cancer Center Hospital, 5-1-1 Tsukiji, Chuo-ku, Tokyo 104-0045, Japan; Department of Head and Neck, Esophageal Medical Oncology, National Cancer Center Hospital, 5-1-1 Tsukiji, Chuo-ku, Tokyo 104-0045, Japan; Department of Pathology and Clinical Laboratories, National Cancer Center Hospital, 5-1-1 Tsukiji, Chuo-ku, Tokyo 104-0045, Japan; Department of Head and Neck Surgery, National Cancer Center Hospital, 5-1-1 Tsukiji, Chuo-ku, Tokyo 104-0045, Japan; Department of Radiation Oncology, National Cancer Center Hospital, 5-1-1 Tsukiji, Chuo-ku, Tokyo 104-0045, Japan; Radiation Safety and Quality Assurance Division, National Cancer Center Hospital, 5-1-1 Tsukiji, Chuo-ku, Tokyo 104-0045, Japan; Department of Radiation Oncology, National Cancer Center Hospital, 5-1-1 Tsukiji, Chuo-ku, Tokyo 104-0045, Japan; Department of Radiation Oncology, National Cancer Center Hospital, 5-1-1 Tsukiji, Chuo-ku, Tokyo 104-0045, Japan; Department of Radiology, Kurume University School of Medicine, 67 Asahimachi, Kurume city, Fukuoka 830-0011, Japan; Department of Radiation Oncology, National Cancer Center Hospital, 5-1-1 Tsukiji, Chuo-ku, Tokyo 104-0045, Japan

**Keywords:** sinonasal undifferentiated carcinoma (SNUC), maxillary sinus, residual tumor, high-dose-rate interstitial brachytherapy boost (HDR-ISBT), radiation therapy (RT)

## Abstract

Sinonasal undifferentiated carcinoma (SNUC) is a highly aggressive and uncommon neoplasm that arises from the mucosa of the nasal cavity or paranasal sinuses. The multidisciplinary approach that includes surgery, radiation therapy (RT), and chemotherapy has been proven to improve survival rates. However, there is no established evidence for the efficacy of further (boost) irradiation following definitive RT in SNUC patients with residual primary tumor. We describe a successful case of a patient with SNUC who had an uncontrolled primary tumor following induction chemotherapy and radical concurrent chemoradiotherapy (CCRT) and underwent a high-dose-rate interstitial brachytherapy (HDR-ISBT) boost. A 75-year-old Japanese woman with unresectable locally advanced SNUC (LA-SNUC) received induction chemotherapy followed by radical CCRT. However, because the residual primary tumor was evident after planned external beam RT, she underwent an HDR-ISBT boost, and the tumor decreased significantly. A complete response (the Response Evaluation Criteria in Solid Tumors, ver. 1.1) was achieved 2 months after brachytherapy, and the patient has been disease-free for 2 years following treatment initiation. In conclusion, an HDR-ISBT boost can be a safe and effective treatment option in patients with residual and inoperable LA-SNUC in the maxillary sinus after initial RT.

## INTRODUCTION

Sinonasal undifferentiated carcinoma (SNUC) is a rare and highly aggressive neoplasm that arises from the mucosa of the nasal cavity or paranasal sinuses, with an estimated incidence of 0.02 per 100 000 [1]. Only 6% of the patients had T1 or T2 lesions at the time of diagnosis, according to the National Cancer Database (NCDB), and the 5-year survival rate was only 42% [2]. Other research has reported that locally advanced SNUC (LA-SNUC) is detected in stage T4 in 71–100% of patients [3, 4]. Although SNUC treatment is still debatable and no consensus has been reached on the best treatment regimen, a combination of surgery, radiation therapy (RT), and/or chemotherapy has been shown to improve survival results compared to one of these treatments alone [2, 5]. Furthermore, some findings showed that induction chemotherapy followed by surgery and/or concurrent chemoradiotherapy (CCRT) can be effective [6–9], and we have reported two successful cases of induction chemotherapy with docetaxel, cisplatin, and fluorouracil (TPF) followed by CCRT for unresectable LA-SNUC [10]. However, to our knowledge, there is no report of additional (boost) irradiation after definitive external beam radiation therapy (EBRT) in SNUC patients with residual primary tumor. Here, we describe a case of a patient with LA-SNUC who had an uncontrolled primary tumor by induction chemotherapy and radical CCRT. The patient was treated with a high-dose-rate interstitial brachytherapy (HDR-ISBT) boost for the primary tumor, followed by nivolumab, resulting in a complete response.

**Fig. 1:**
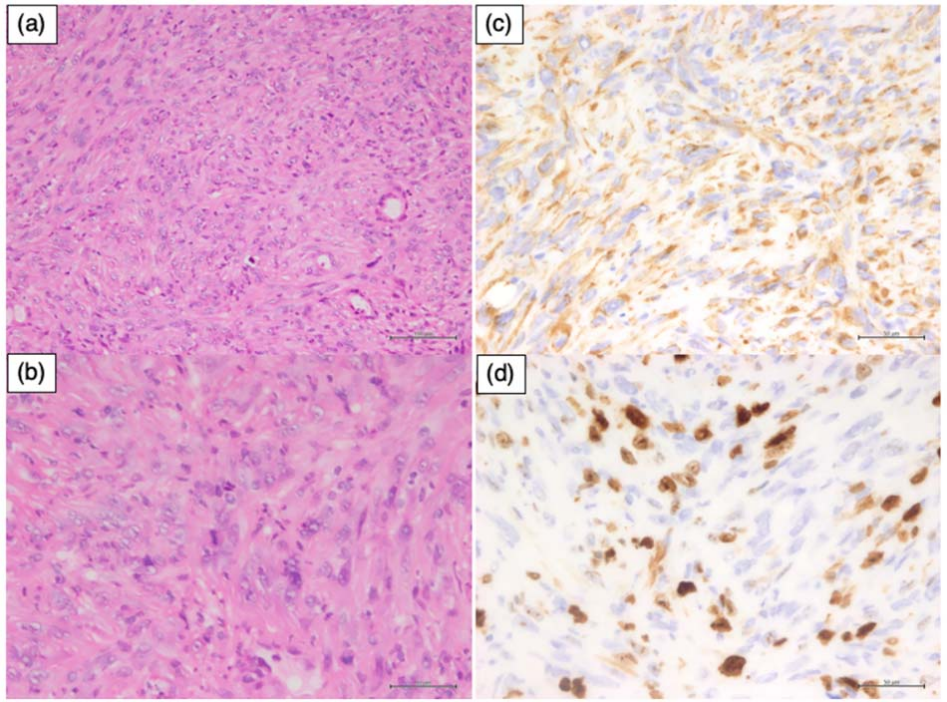
Pathological findings. (a) The tumor showed polygonal epithelial cells without squamous and glandular features (hematoxylin–eosin [H&E], low magnification). (b) (H&E, high magnification). (c) Tumor cells were weakly positive for cytokeratin (Cytokeratin AE1/3 immunohistochemistry). (d) The Ki-67 proliferation index was around 30%. Sections of H&E staining showed polygonal epithelial cells with fine chromatin and mitoses without evidence of squamous or glandular differentiation. The tumor exhibited weak cytoplasmic positivity for cytokeratin AE1/AE3, CAM5.2, INI1/BAF47, and Vimentin while being negative for SMA, CK7, CK5/6, p40, SOX10, CD45/LCA, CD30, CD34, EMA, NUT, and Synaptophysin. The Ki-67 proliferation index was 30%, leading to the final diagnosis of SNUC.

## CASE PRESENTATION

**Fig. 2:**
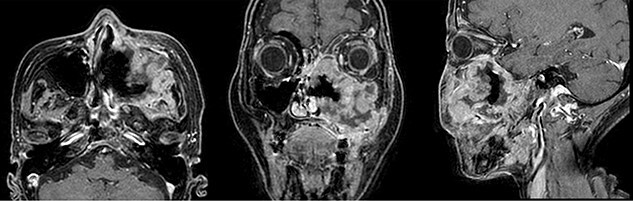
Magnetic resonance images with contrast media before treatment. The tumor extended superiorly into the orbit of the left eye and a rotundum foramen, anteriorly to the subcutaneous tissue that destroyed the maxillary sinus wall, and posteriorly into the left infratemporal fossa and the pterygopalatine fossa. She was diagnosed with T4bN0M0 left maxillary sinus cancer; LA-SNUC.

**Fig. 3:**
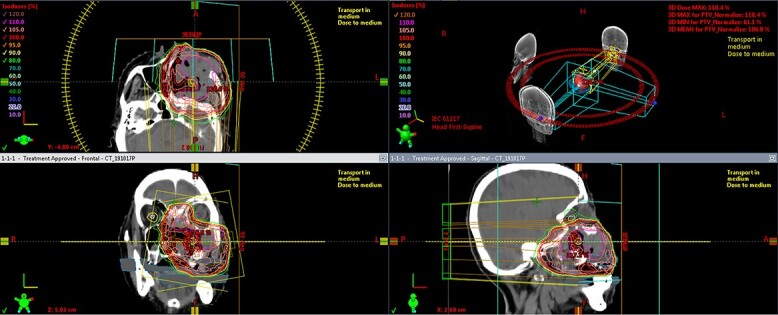
A dose distribution of VMAT for locally advanced SNUC of left maxillary sinus cancer.

A 75-year-old Japanese woman with a large epistaxis was diagnosed with T4bN0M0 left maxillary sinus cancer (LA-SNUC) by various examinations (Figs 1 and 2). The tumor was considered unresectable due to the involvement of the foramen rotundum, induction chemotherapy with TPF chemotherapy (docetaxel, 70 mg/m^2^, day 1; cisplatin, 70 mg/m^2^, day 1; fluorouracil, 750 mg/m^2^, days 1 to 5) was started 3 weeks after the onset of massive epistaxis, with a dose reduced to 80% due to moderate renal dysfunction (CCr = 48.6 ml/min) and copious epistaxis caused by myelosuppression. However, computed tomograms (CT scans) after the completion of the second TPF cycle, which was routinely performed just before the next cycle to determine the treatment effect, revealed that the tumor had enlarged compared to the pretreatment level by more than 20% and was considered a progressive disease (the Response Evaluation Criteria in Solid Tumors, version 1.1). Therefore, she received subsequent EBRT using volumetric modulated arc therapy (VMAT) up to 70 Gy in 35 fractions (Fig. 3). However, at the time of 64 Gy irradiation, MR images showed that most of the tumor still remained, and the contrast enhancement 

**Fig. 4:**
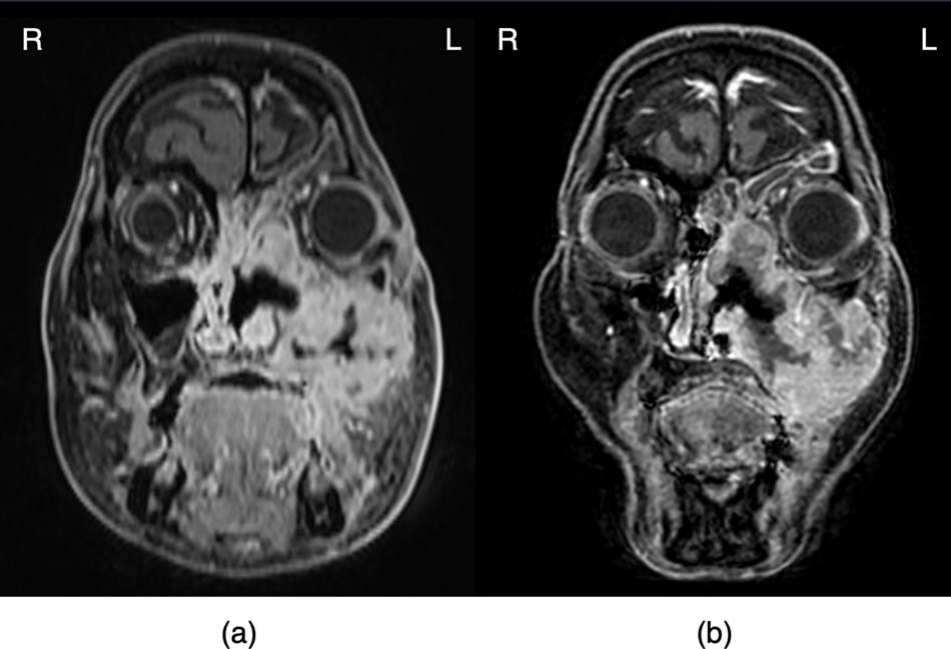
Comparison of magnetic resonance images (a) before treatment and (b) before the completion of CCRT. These images revealed that most of the tumor still remained before the completion of CCRT.

was confirmed, so it was judged that the tumor could not be controlled by planned EBRT and chemotherapy alone (Fig. 4). Therefore, 1 week after the completion of CCRT, she received an HDR-ISBT boost of 16 Gy in four fractions to control the primary tumor, which resulted in a significant reduction in residual tumor volume. The detailed procedure for an HDR-ISBT boost was as follows (Fig. 5): Considering that a single needle applicator could cover an area of approximately 5 mm in radius with a high dose (approximately 100% of the target dose), volume-implant with four parallel planes at 1 cm intervals was performed so that the needles were equally distributed to cover the entire residual tumor. Nine needle applicators were inserted percutaneously under local anesthesia and sedation with the patients in the supine position and guided by a real-time lateral fluoroscopic X-ray image for needle depth. A simulation CT was performed with a large-bore CT (Aquilion® LB, Canon, Tokyo, Japan), and image-guided brachytherapy planning was performed based on CT scans with a slice interval of 2 mm. Treatment planning was carried out with the brachytherapy planning system (Oncentra® Elekta, Veenendaal, The Netherlands). Clinical tumor volume (CTV) was defined based on the CT scans taken after needle insertion with patients in the supine position, with information derived from MR images just before needle insertion. Reference points were established on the surface of the CTV and 4 Gy was delivered to those points. Dose calculation was performed by geometrical optimization followed by a manual graphical modification to cover the CTV with the 100% prescribed isodose line of 4 Gy while avoiding the hyper-dose sleeves, representing 200% of the prescribed reference dose, as small as possible and keeping the dose to the left optic nerve to a minimum. The combined dose of EBRT and HDR-ISBT was calculated according to the linear-quadratic dose-effect model of the equivalent dose in 2 Gy fraction (EQD2) [11–13], using the following formula: EQD2 = D (d + α/β)/(2 + (α/β)), where D is the total prescription dose, d is the fractional dose, and α/β is the alpha/beta ratio; it is 10 Gy for head and neck tumors and 3 Gy for organs at risk (OARs). In this case, the minimum dose of EQD2 that covered 90% of the CTV was 85.4 Gy of EQD_2_ (α/β = 10 Gy). On the other hand, the dose prescribed for 0.1 cm^3^ of the left lens (D0.1 cm^3^) was 26.9 Gy of EQD2, for D0.1 cm^3^ of the left eyeball was 86.1 Gy of EQD2, and for D0.1 cm^3^ of the left optic nerve was 54.0 Gy of EQD2 (α/β = 3 Gy). An HDR-ISBT boost was performed twice daily at a 6 hour intervals, and needles were fixed to the skin with sutures and remained in place until HDR-ISBT was completed for 3 days with grade 1 stomatitis (the National Cancer Institute Common Terminology Criteria for Adverse Events, version 5.0). However, despite the gradual shrinkage and disappearance of the maxillary sinus tumor soon after these treatments, bilateral cervical and supraclavicular lymph node metastases appeared, indicating that the patient had become resistant to chemotherapy, and 2 weeks after the completion of brachytherapy, nivolumab (240 mg/body, day 1) was started at 2 week intervals.

## RESULTS

Two months after brachytherapy, CT scans revealed a complete response, and she has been disease-free for 2 years after starting salvage treatment (Fig. 6).

## DISCUSSION

EBRT is generally the mainstay of therapy for maxillary sinus cancer, and several studies suggested that in SNUC, there may be a dose–response relationship and that at least 60 Gy is needed [14–16]. In the present case, however, primary tumor control was insufficient despite the administration of induction chemotherapy followed by radical CCRT with a prescribed dose of 70 Gy. On the other hand, brachytherapy is rarely used, even for boost irradiation, but it is technically feasible and there are few OARs in the case of maxillary cancer treatment.

Regarding the radical treatment of SNUC, previous studies have reported that a multidisciplinary approach including surgery, RT, and/or chemotherapy can be effective. However, to our knowledge, there is no detailed report on boost irradiation when initial treatment, including RT, failed and was determined to be inoperable. Distant metastasis is rare in SNUC [17], and therefore, we believe that local control should be the main priority during initial treatment. We chose to deliver an HDR-ISBT boost to achieve tumor control, considering the absence of critical organs, except the optic nerve, which hinders additional irradiation and the tolerable dose of OARs and the patient’s quality of life. We prescribed a dose similar to that in previous cases of pharyngeal malignancies in which disease control was achieved with an HDR-ISBT boost of 4 Gy per fraction after full-dose radical CCRT [18–20]. The equivalent dose in the 2 Gy fraction of the maximum dose for her left optic nerve was 58.2 Gy of EQD2 (α/β = 3 Gy), which was considered acceptable and below the tolerable dose to the optic nerve [21]. EBRT often cannot safely deliver more than 70 Gy to eradicate residual tumors with previous RT due to the high risk of severe toxicities, HDR-ISBT has the intrinsic physical characteristic of having a steep dose gradient, and several reports suggested the efficacy and safety of HDR-ISBT for patients with maxillary sinus malignancies who had a history of RT [22–24].

As an alternative to an HDR-ISBT boost, intraoperative irradiation, stereotactic irradiation, and particle therapy may be useful. Particle radiotherapy, in particular, has physical characteristics, such as the fact that the dose rapidly decreases after concentrated energy is given to tissues near the far end (Bragg peak) and that the width of the irradiation field (penumbra) is much narrower than that of X-ray therapy, which allows high doses to be prescribed to lesions while reducing the doses to the surrounding organs, making it suitable for irradiation in the abdominal cavity and pelvis, where many vital organs are located. However, in a comparative study of brachytherapy, intensity-modulated photon therapy, and intensity-modulated proton beam therapy, Georg *et al.* reported that the entrance dose of photon beams and the plateau dose of proton beams increase the contributing dose to the OARs [25]. The same may be true for carbon ion beams, which show a Bragg curve similar to that of protons. In addition, particle beams also have other disadvantages, such as the need for preparation time and the high cost of treatment. On the other hand, HDR-ISBT, although an invasive treatment, is considered to provide an ideal dose distribution while reducing the dose to the OARs since the placement time of the radioactive source can be easily adjusted, the margin for movement can be ignored, and it exhibits a steep dose gradient.

**Fig. 5:**
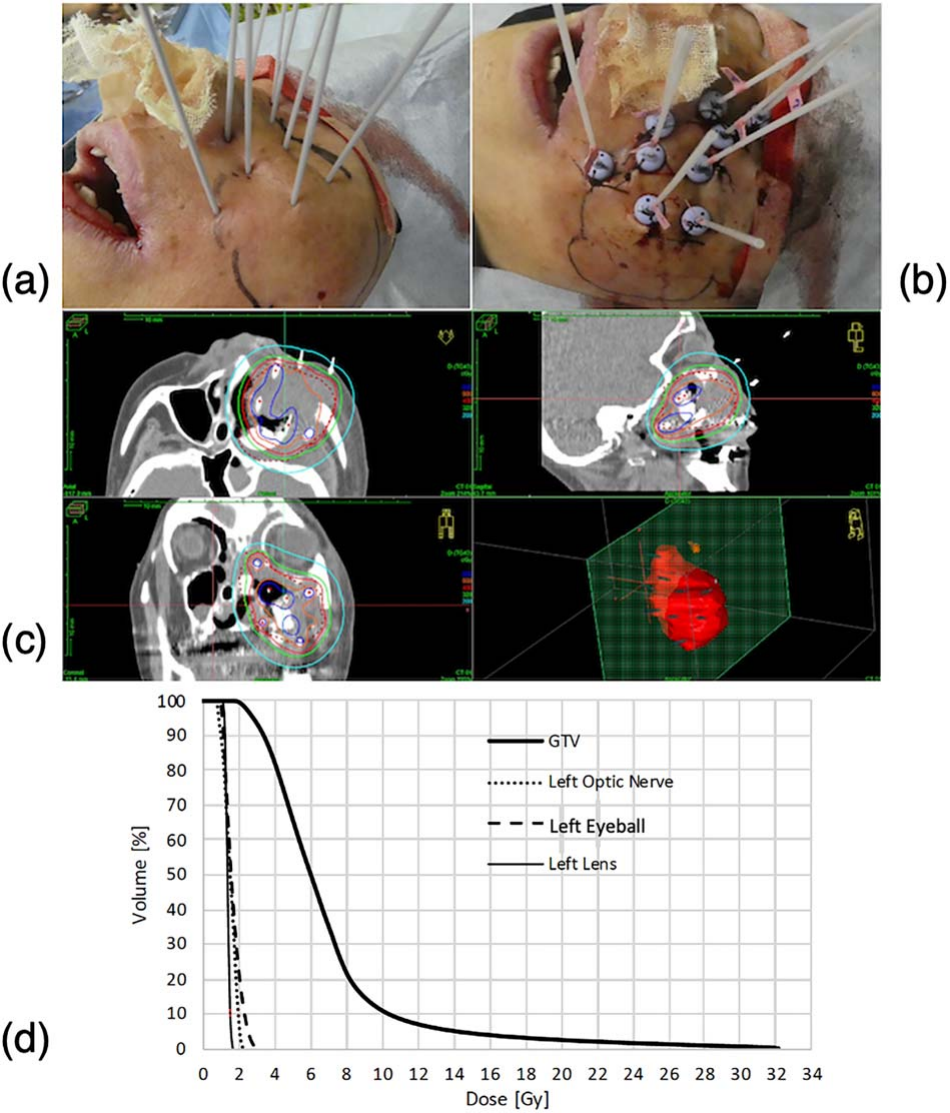
The procedure of an HDR-ISBT boost. (a) Nine needle applicators were inserted percutaneously under local anesthesia with the patients in the supine position and guided by fluoroscopic X-ray image. (b) The needles were attached to the skin with sutures and remained in place until HDR-ISBT was complete. (c) Isodose distribution in the brachytherapy planning system with 100% (red) and 200% (blue) isodose lines. (d) A dose volume histogram of the CTV, the left lens, the left eyeball, and the left optic nerve.

**Fig. 6:**
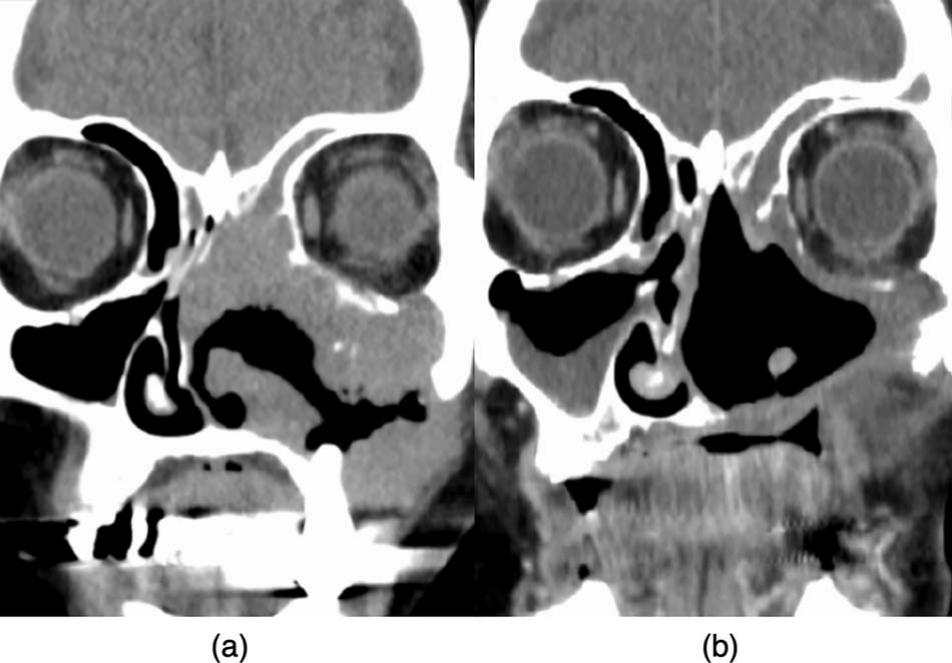
Comparison of CT scans (a) before treatment and (b) after an HDR-ISBT boost. These images revealed a complete response (the Response Evaluation Criteria in Solid Tumors, version. 1.1).

HDR-ISBT could also be used to treat other diseases like pharyngeal or nasal cancer [18–20, 26]. For the nasal cavity, in particular, an HDR-ISBT boost is a promising treatment option because, unlike the pharynx, it has the anatomical advantage of not having to worry about the risk of carotid artery rupture due to ulcer formation associated with tumor shrinkage after irradiation or the treatment procedure.

The observation period was insufficient to ascertain whether local control had been achieved. While an HDR-ISBT boost may have played a significant role in the 2 year local control, it is also possible that nivolumab, which was initiated for recurrence outside the irradiated field, played a role, and thus this case cannot be considered solely due to an HDR-ISBT boost. Despite these limitations, we reported this case because we believed it was a novel method for safely administering high doses locally and the patient remained recurrence-free for 2 years.

In conclusion, an HDR-ISBT boost can be an effective and safe treatment method to deliver a high radiation dose to residual SNUC after initial CRT, due to its physical characteristic of a steep dose gradient. The authors will continue to employ this technique, when feasible, for a variety of head and neck tumors that are not controlled after initial radical EBRT.

## CONFLICT OF INTEREST

H.I. received a research grant from HekaBio, CICS, and Elekta KK, received consulting fees from HekaBio, and lecture fees from Varian, Itochu, CICS, and Himedic, outside the submitted work. J.I. received a research grant from Elekta, and received a consulting fees from AlphaTAU, HekaBio, and Palette Science, outside the submitted work. T.K. received lecture fees from AstraZeneca, outside the submitted work. The other authors have nothing to disclose.
